# Use of antenatal and delivery care services and their association with maternal and infant mortality in rural India

**DOI:** 10.1038/s41598-022-20951-9

**Published:** 2022-10-03

**Authors:** Rajesh Kumar Rai, Anamitra Barik, Abhijit Chowdhury

**Affiliations:** 1 Department of Economics, University of Göttingen, 37073 Göttingen, Germany; 2 Centre for Modern Indian Studies, University of Göttingen, 37073 Göttingen, Germany; 3 Society for Health and Demographic Surveillance, Suri, West Bengal 731101 India; 4 Department of Global Health and Population, Harvard T H Chan School of Public Health, Boston, MA 02115 USA; 5 Suri District Hospital, Suri, West Bengal 731101 India; 6 School of Digestive and Liver Disease, Institute of Post Graduate Medical Education and Research, Kolkata, West Bengal 700020 India; 7 John C Martin Centre for Liver Research and Innovations, Indian Institute of Liver and Digestive Sciences, Liver Foundation West Bengal, Kolkata, West Bengal 700150 India

**Keywords:** Health services, Public health

## Abstract

Optimum use of antenatal care (ANC) and delivery care services could reduce morbidity and mortality among prospective mothers and their children. However, the role of ANC and delivery services in prevention of both maternal and child mortality is poorly understood, primarily because of dearth of prospective cohort data. Using a ten-years population-based prospective cohort data, this study examined the use of ANC and delivery services and their association with maternal and infant mortality in rural India. Descriptive statistics were estimated, and multivariable logistic regression modelling was used to attain the study objective. Findings revealed that consumption of ≥ 100 iron-and-folic acid (IFA) tablet/equivalent syrup during pregnancy had a protective association with maternal and infant mortality. Lack of maternal blood group checks during pregnancy was associated with increased odds of the death of infants. Caesarean/forceps delivery and delivery conducted by untrained personnel were associated with increased odds of maternal mortality. Findings from this study reemphasizes on increasing coverage and consumption of IFA tablets/equivalent syrup. Improved ANC and delivery services and increased uptake of all types of ANC and delivery care services are equally important for improvement in maternal and child survival in rural India.

## Introduction

Despite an estimated reduction in both maternal and child deaths since 1990, India still accounts for the highest number of maternal deaths^1^ and child mortality^2^ in the world. To achieve target 3.1 of the United Nations’ Sustainable Development Goals (SDGs), which aims to reduce the global maternal mortality ratio (MMR) to less than 70 per 100,000 live births, India must prioritize their effort to reduce its current MMR, which is 113 per 100, 000 live births as estimated in 2016–2018^3^. India’s infant mortality rate (per 1000 live birth) has decreased from 37/1000 in 2015 to 30/1000 in 2019^4^; however, much is yet to be done to help achieve the target of reducing under-five mortality set in SDG (Target 3.2). To reap the benefit of key interventions designed to prevent maternal and child mortality, it is crucial to strengthen the continuum of reproductive, maternal, newborn and child health care (RMNCH) services^5,6^ proposed by the World Health Organization (WHO). A continued focus on RMNCH is essential to address the considerable burden of maternal and child mortality from unwanted pregnancies; high maternal, newborn, and child mortality and stillbirths; high rates of undernutrition; and frequent communicable and noncommunicable diseases^7^. Within the continuum of RMNCH services, antenatal care (ANC) and delivery care are keys to provide a platform for crucial maternal and child health care functions including health promotion, screening and diagnosis, and disease prevention^8^.

Within the literature on the role of appropriate ANC and delivery care in improving maternal and child health, a systematic review^9^ which assessed the impact of different types of death audits and reviews in reducing maternal, perinatal and child mortality, highlighted the importance of using healthcare facilities to reduce maternal and child mortality. This review is suggestive of the protective effect of appropriate ANC and delivery care use in mitigating maternal and child mortality. Another review^10^ reiterated the importance of skilled delivery and facility-based services for maternal and newborn care in low- and middle-income countries (LMICs). A systematic review and meta-analysis^11^ conducted for African countries identified the relative advantage of having ANC to give birth in health facilities. Two studies^12,13^ of pooled data from LMICs were also supported improvement in the quality of ANC to reduce the risk of neonatal mortality. Few recent studies ^14–16^ also concluded that use of ANC services are critical factors in reducing the risk of infant and neonatal mortality.

India’s Ministry of Health and Family Welfare has adopted and implemented the WHO’s ANC^8^ and delivery care^17^ guidelines as part of the Reproductive, Maternal, Newborn Child plus Adolescent Health (RMNCH + A)^5^ program. RMNCH + A runs under the ambit of the National Health Mission, a flagship programme of the Ministry of Health and Family Welfare, Government of India. ANC and delivery care services are part of the 24 × 7 basic and comprehensive obstetric care services operationalized by the sub-centres (the lowest level of health care facility), Primary Health Centres, Community Health Centres and District Hospitals. As estimated in the 2019–2021 National Family Health Survey, only 58.1% women were reported to have made at least four ANC visits for their recent pregnancy in the preceding five years of the survey whereas 89.4% of deliveries were conducted by skilled health personnel^18^.

To date, a range of studies have documented various reasons of poor coverage and uptake of ANC services in India^19–21^, whereas an appreciable improvement in safe delivery care (delivery conducted by skilled personnel) was registered between 2015–2016 and 2019–2021^18^. Select recent studies have empirically demonstrated that maternal mortality could be mitigated by addressing socio-economic barriers and by encouraging appropriate use of the healthcare infrastructure in India^22–25^. Although several studies have documented the relation between ANC uptake and child mortality ^26,27^, studies which assess the relationship between the use of ANC services and maternal mortality are scarce. A handful of studies^28,29^ which attempted to do so did not have the follow-up data to analyse the use of ANC services and its relationship with maternal death. Similarly, safe delivery care was found to be protective for neonatal mortality and infant mortality in India ^30–32^ whereas empirical analysis on linkages between safe delivery and maternal mortality is rare. To fill this research gap, this study uses a ten-years population-based prospective cohort data on pregnancies available from a health and demographic surveillance system located in rural India, to examine if the use of various components of ANC and delivery care services are associated with maternal and infant mortality in India.

## Methods

### Study setting and dataset

**Figure 1:**
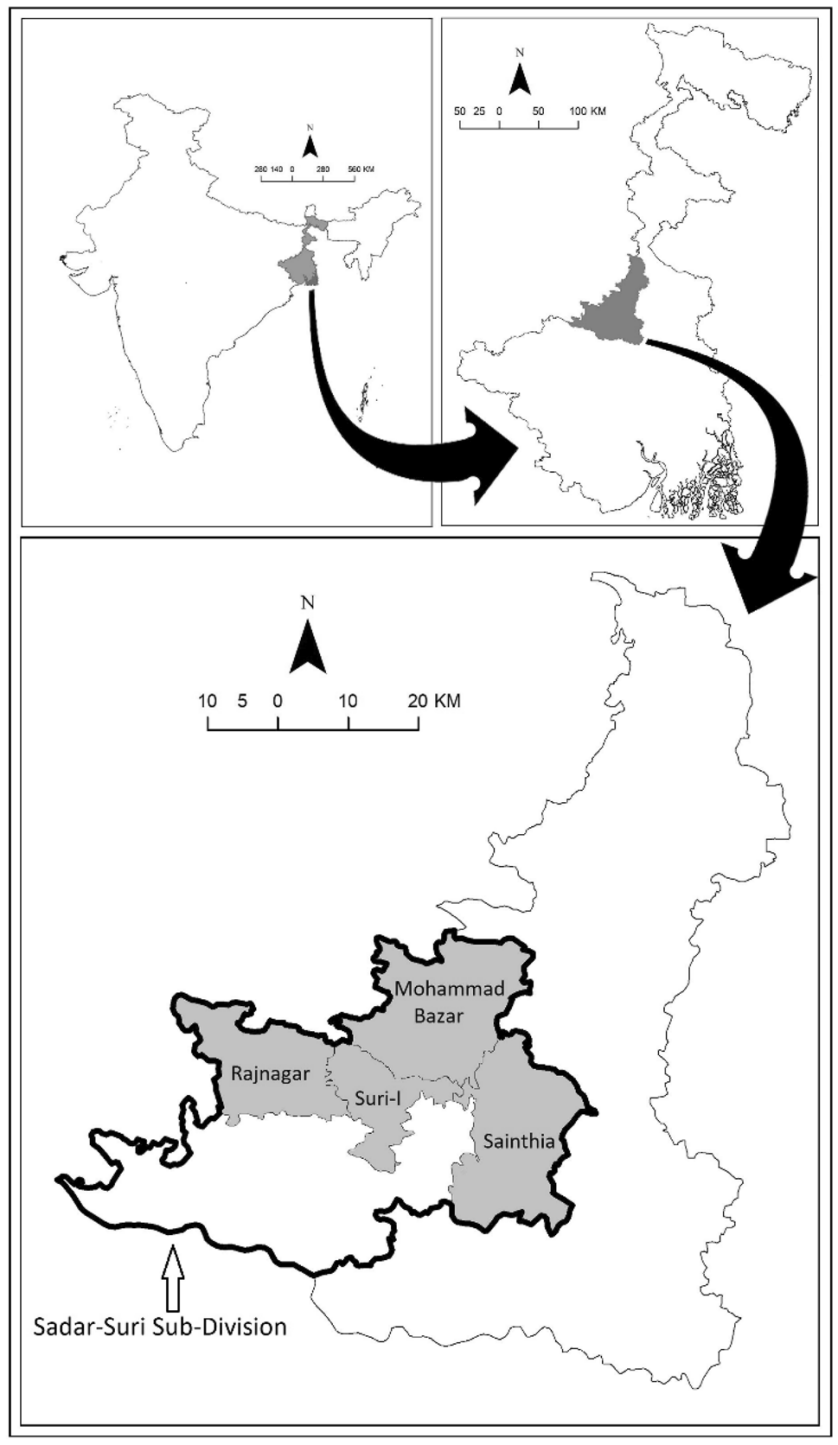
Study area of the Birbhum Population Project, Society for Health and Demographic Surveillance. Figure was developed using ArcGIS Software, Release 10: https://www.arcgis.com.

For this study, all aspects of methods were followed in accordance with relevant guidelines, regulations, and international practice of conducting scientific research. Data utilized for the study were retrieved from the Birbhum Population Project (BIRPOP), a health and demographic surveillance system (HDSS), located in the Birbhum district of eastern state of West Bengal in India^33^. Established under the ambit of Society for Health and Demographic Surveillance (https://www.shds.co.in) in 2008, BIRPOP is governed and financially supported by the Department of Health and Family Welfare, the State Government of West Bengal. BIRPOP’s survey area covers four administrative blocks, namely Mohammad Bazar, Rajnagar, Sainthia and Suri-1 (Fig. 1). The study area of BIRPOP represents nearly 16% of the total population of Birbhum district (as per the Census of India, 2011). Predominantly rural, the Birbhum district is recognized to have poor human development indicators by the NITI *Aayog*, an apex policy thinktank of the Government of India^34^. The primary objective of BIRPOP is to undertake research and gather evidence to inform the state- and national public health policies. In 2009, helped by experts from academic institutions, BIRPOP selected a self-weighted sample of 59,395 individuals from 13,053 households spread across 351 villages, and undertook various research programmes including continuous collection of information on vital events (birth, death, marriage, and migration)^33^.

To date, BIRPOP has carried out three rounds (2008–2009, 2012–2013 and 2017–2018) of follow-up surveys on various health and demographic indicators, and few cross-sectional surveys were also undertaken. A detailed overview of the BIRPOP-HDSS can be found in its published profile report^33^.

Tracking and gathering information on pregnancies is one of the ongoing flagship surveys of BIRPOP^33^. Although 13,053 households are the primarily sampled as part of the BIRPOP-HDSS, it collects information on all pregnancies reported in the BIRPOP survey area. This study uses pregnancies recorded between January 01, 2012, and December 31, 2021, totalling a duration of 10 years. During this 10 years of pregnancy tracking, BIRPOP collected information on 42,040 pregnancies with complete information on ANC and delivery care, which was included in this study, whereas 37,183 children were included to analyse infant mortality. Pregnancies were followed from the day of reporting conception of pregnancy till one year after the delivery date. Collecting information on pregnancies is part of the regular survey activity of BIRPOP, and depending on the information collated, the database is updated continuously.

### Tracking of pregnancies and definition of maternal and infant mortality

The flagship programme of tracking pregnancies in BIRPOP was launched in 2012, before the framework of RMNCH strategy was initiated by the Government of India^35^. BIRPOP designed a semi-structured questionnaire to follow and gather information on various components of ANC and delivery care services. Tracking of ANC and delivery care services and recording the obtained information is a mammoth endeavour but having 44 full-time salaried surveyors (individuals who are responsible for collecting data from each household) and stringent data collection and recording protocol help BIRPOP run this task successfully. Over 98% surveyors are females with at least an undergraduate degree (Bachelor’s degree), and they are proficient in the local language (Bengali or Santhali). Surveyors of BIRPOP are recruited from the communities being surveyed, which helps to track pregnancies in the survey area. After recruitment, the surveyors underwent rigorous training for collecting information on ANC and delivery care services by experienced physicians and social scientists. With their satisfactory performance in collecting information on RMNCH, they are allowed to conduct household surveys on pregnancy tracking. It is worth emphasizing that since the surveyors of BIRPOP are salaried employees, it is mandatory for them to pay a visit to every household at least once in a month, and while interacting with the household members during their visit, they come to know if any married woman in a household has conceived. If conception is reported, the information on ANC is recorded time to time, which is followed by maternal delivery care and health outcomes of pregnancy (if the mother was healthy or experienced complications or died) and child health outcomes (if it was a live birth, if the newborn was underweight). If the pregnancy resulted into abortion or still-birth, this information is recorded as well. It is worth mentioning that if any woman reported having moved to a different place after conceiving or delivering the baby, all related information on pregnancy tracking survey is collected via telephonic interviews and the information recorded is also confirmed by the family member. If there is any discrepancy found once the surveyor meets the woman upon her return, the pregnancy tracking database is amended accordingly.

BIRPOP defines maternal and infant mortality as per the guideline developed by the WHO. A maternal death is “female death from any cause related to or aggravated by pregnancy or its management (excluding accidental or incidental causes) during pregnancy and childbirth or within 42 days of termination of pregnancy, irrespective of the duration and site of the pregnancy”, and infant mortality is the death of a child before reaching the age of one year^4^.

### Predictors of maternal and infant mortality

While ANC and delivery care received during pregnancy were considered as the primary potential predictors of maternal and infant mortality, few other predictor variables were also developed using the pregnancy tracking database. Of the components of ANC, variables representing demographic characteristics are age at conception in completed years (< 18, 18–21, and ≥ 21) and gravida (1, 2, 3, and ≥ 4); whereas information on pregnancy registration (with primary health care centre, or with other establishments); whether blood pressure, weight, blood group, haemoglobin level, urine sugar, and urine protein were checked during the pregnancy; number of iron-and-folic-acid (IFA) tablet/equivalent syrup consumed (< 100 and ≥ 100) during pregnancy; and total number of ANC visits (< 4 and ≥ 4). A pregnancy registered with “other establishments” refers to all types of private clinics including pregnancy registration with informal healthcare providers and sub-centre. Marriage before 18 years of age is defined as child marriage in India, and it is illegal^36^. Child marriage could lead to pregnancy immediately after marriage which could lead to poor maternal and child health outcomes^36^. For healthy pregnancy outcome, WHO recommends ≥ 100 IFA consumption during pregnancy, and encourages at least four ANC visits^35^.

On delivery care, information on the place of delivery (health care facility, and non-health care facility), the type of delivery (normal and caesarean/forceps) and the personnel who assisted in delivery (trained personnel and untrained personnel) were used as potential predictors of maternal and infant mortality. As predictors of infant mortality, sex of the child (male and female) and information on birthweight status (low birthweight and normal) were also considered. Babies weighing < 2.5 kg at birth were defined as having low birthweight^37^.

### Statistical analysis

Bivariate analysis was used to estimate the distribution of sample used for the study and the proportion of pregnancy resulted into maternal and infant mortality, by select background characteristics. Univariable binary (coded “1” if maternal or infant mortality, otherwise “0”) logistic regression was run to examine the association between each predictor variable and maternal and infant death, whereas a multivariable binary logistic regression model was developed to understand the net association between predictor variables and outcome events—maternal and infant mortality. Variance inflation factors (VIF) were estimated to assess the presence of multicollinearity among the predictors^38^. The estimated VIF were < 5, indicating low probability of multicollinearity. The statistical software, Stata, version 14^39^ was used to analyse the data, and a *p* value (level of significance) of < 0.05 (two-tailed) obtained from the logistic regression modelling are the focus of discussion.

### Ethics declarations

Ethical approval was obtained from the institutional ethics review board of BIRPOP-HDSS, appointed by the chairperson of the SHDS. Informed written consent was obtained from the respondent with a declaration from SHDS that individual identifiers will be removed if dataset is made public for the use by researchers.

## Results

A description of the sample distribution is presented in Table 1. Of the total (n = 42,040) pregnancies included for analysing maternal mortality, nearly 8.7% were conceived before the age of 18 years, and 47.4% women were with their first gravida. Nearly 86% of pregnancies were delivered at a health facility; 79.8% pregnancies were delivered normal whereas caesarean/forceps delivery were 20.2%. Only 34.9% of women consumed ≥ 100 iron-and-folic-acid tablets/ equivalent syrup during their pregnancies. Of the total 37,183 children, 51.7% were male. Nearly 19.5% of children weighted < 2.5 kg at their birth.

**Table 1:** Sample distribution of total pregnancies used to measure maternal and infant mortality, by select background characteristics.

	Maternal mortality n (%)	Infant mortality n (%)
**Age at conception**		
< 18	3659 (8.7)	3196 (8.6)
18–21	19,447 (46.3)	17,225 (46.3)
≥ 22	18,934 (45.0)	16,762 (45.1)
**Gravida**		
1	19,921 (47.4)	17,664 (47.5)
2	15,631 (37.2)	13,837 (37.2)
3	4891 (11.6)	4310 (11.6)
≥ 4	1597 (3.8)	1372 (3.7)
**Pregnancy registered with**		
Primary Healthcare Centre	33,770 (80.3)	30,057 (80.8)
Other establishment	8270 (19.7)	7126 (19.2)
**Blood pressure was checked**		
Yes	41,376 (98.4)	36,916 (99.3)
No	664 (1.6)	267 (0.7)
**Weight was checked**		
Yes	41,420 (98.5)	36,920 (99.3)
No	620 (1.5)	263 (0.7)
**Blood group was checked**		
Yes	25,281 (60.1)	23,049 (62.0)
No	16,759 (39.9)	14,134 (38.0)
**Haemoglobin level was checked**		
Yes	40,220 (95.7)	36,040 (96.9)
No	1820 (4.3)	1143 (3.1)
**Urine sugar was checked**		
Yes	35,782 (85.1)	32,215 (86.6)
No	6258 (14.9)	4968 (13.4)
**Urine protein was checked**		
Yes	35,165 (83.7)	31,663 (85.2)
No	6875 (16.4)	5520 (14.9)
**Number of antenatal care visit**		
< 4	28,773 (68.4)	24,648 (66.3)
≥ 4	13,267 (31.6)	12,535 (33.7)
**Place of delivery**		
Healthcare facility	36,144 (86.0)	32,650 (87.8)
Non-healthcare facility	5896 (14.0)	4533 (12.2)
**Type of delivery**		
Normal	33,546 (79.8)	29,738 (80.0)
Caesarean/forceps	8494 (20.2)	7445 (20.0)
**Delivery conducted by**		
Trained personnel	39,777 (94.6)	35,934 (96.6)
Untrained personnel	2263 (5.4)	1249 (3.4)
**Number of iron-and-folic acid consumption**		
< 100	27,355 (65.1)	23,135 (62.2)
≥ 100	14,685 (34.9)	14,048 (37.8)
**Sex of the child**		
Male	Na	19,211 (51.7)
Female	Na	17,972 (48.3)
**Birthweight status**		
Low birthweight	Na	7250 (19.5)
Normal	Na	29,933 (80.5)
**Overall**	42,040 (100.0)	37,183 (100.0)

Percentage distribution may not add to 100 due to rounding.

*n* sample, *na* not applicable.

Burden and factors associated with maternal deaths is presented in Table 2. Overall, 0.088% (95% confidence interval or CI: 0.064–0.121%) maternal death was estimated. Similar to the univariable model, multivariable model revealed that caesarean/forceps delivery and delivery conducted by untrained professionals were associated with increased odds of maternal deaths—odds ratio (OR): 6.61, 95% CI: 3.19–13.31) *p* < 0 0.001 and OR: 3.08 (95% CI: 1.02–9.28), *p* = 0.046, respectively. A protective association between ≥ 100 iron-and-folic-acid tablet/ equivalent syrup consumption and maternal deaths was observed (OR: 0.19, 95% CI: 0.06–0.54, p = 0.002).

**Table 2:** Factors associated with maternal mortality.

	Maternal mortality	Univariable model	Multivariable model
	% (95% CI)	OR (95% CI), p	OR (95% CI), p
**Age at conception**			
< 18	0.082 (0.026–0.254)	1.00 (referent)	1.00 (referent)
18–21	0.046 (0.024–0.089)	0.56 (0.15–2.09), 0.391	0.57 (0.15–2.13), 0.400
≥ 22	0.132 (0.089–0.195)	1.61 (0.49–5.34), 0.435	1.52 (0.39–5.96), 0.550
**Gravida**			
1	0.070 (0.042–0.119)	1.00 (referent)	1.00 (referent)
2	0.090 (0.053–0.151)	1.27 (0.61–2.67), 0.521	0.90 (0.37–2.16), 0.811
3	0.143 (0.068–0.300)	2.04 (0.82–5.05), 0.124	1.22 (0.41–3.62), 0.720
≥ 4	0.125 (0.031–0.500)	1.78 (0.40–7.85), 0.445	1.05 (0.21–5.31), 0.955
**Pregnancy registered with**			
Primary Healthcare Centre	0.095 (0.067–0.134)	1.00 (referent)	1.00 (referent)
Other establishment	0.060 (0.025–0.145)	0.64 (0.25–1.64), 0.350	0.49 (0.18–1.32), 0.159
**Blood pressure was checked**			
Yes	0.087 (0.063–0.121)	1.00 (referent)	1.00 (referent)
No	0.151 (0.021–1.063)	1.73 (0.24–12.65), 0.588	1.19 (0.03–53.78), 0.927
**Weight was checked**			
Yes	0.087 (0.063–0.120)	1.00 (referent)	1.00 (referent)
No	0.161 (0.023–1.137)	1.86 (0.25–13.57), 0.542	1.41 (0.03–58.32), 0.855
**Blood group was checked**			
Yes	0.071 (0.045–0.113)	1.00 (referent)	1.00 (referent)
No	0.113 (0.072–0.178)	1.59 (0.84–3.04), 0.157	1.59 (0.79–3.21), 0.197
**Haemoglobin level was checked**			
Yes	0.087 (0.062–0.121)	1.00 (referent)	1.00 (referent)
No	0.110 (0.027–0.438)	1.26 (0.30–5.26), 0.748	1.15 (0.17–7.87), 0.889
**Urine sugar was checked**			
Yes	0.095 (0.068–0.133)	1.00 (referent)	1.00 (referent)
No	0.048 (0.015–0.149)	0.50 (0.15–1.64), 0.256	0.22 (0.02–2.21), 0.200
**Urine protein was checked**			
Yes	0.094 (0.067–0.132)	1.00 (referent)	1.00 (referent)
No	0.058 (0.022–0.155)	0.62 (0.22–1.75), 0.366	1.14 (0.17–7.74), 0.896
**Number of antenatal care visit**			
< 4	0.101 (0.070–0.145)	1.00 (referent)	1.00 (referent)
≥ 4	0.060 (0.030–0.121)	0.60 (0.27–1.31), 0.198	0.72 (0.32–1.64), 0.438
**Place of delivery**			
Healthcare facility	0.089 (0.063–0.125)	1.00 (referent)	1.00 (referent)
Non-healthcare facility	0.085 (0.035–0.204)	0.96 (0.37–2.46), 0.929	0.57 (0.17–1.90), 0.364
**Type of delivery**			
Normal	0.051 (0.032–0.082)	1.00 (referent)	1.00 (referent)
Caesarean/forceps	0.235 (0.152–0.365)	4.65 (2.44–8.89), < 0.001	6.51 (3.19–13.31), < 0.001
**Delivery conducted by**			
Trained personnel	0.078 (0.055–0.111)	1.00 (referent)	1.00 (referent)
Untrained personnel	0.265 (0.119–0.589)	3.41 (1.42–8.18), 0.006	3.08 (1.02–9.28), 0.046
**Number of iron-and-folic acid consumption**			
< 100	0.121 (0.086–0.170)	1.00 (referent)	1.00 (referent)
≥ 100	0.027 (0.010–0.073)	0.23 (0.08–0.64), 0.005	0.19 (0.06–0.54), 0.002
**Overall**	0.088 (0.064–0.121)		

*CI* confidence interval, *OR* odds ratio, *p* level of significance.

The predictors of infant mortality are presented in Table 3. Overall, 2.33% (95% CI: 2.18%-2.48%) infant deaths were estimated. In both univariable and multivariable models, maternal blood group check-up and ≥ 100 iron-and-folic-acid tablet/ equivalent syrup consumption had a protective association with infant deaths.

**Table 3:** Factors associated with infant mortality.

	Infant mortality	Univariable model	Multivariable model
	% (95% CI)	OR (95% CI), p	OR (95% CI), p
**Age at conception**			
< 18	3.19 (2.64–3.86)	1.00 (referent)	1.00 (referent)
18–21	2.40 (2.19–2.64)	0.75 (0.60–0.93), 0.009	0.86 (0.69–1.08), 0.197
≥ 22	2.08 (1.88–2.31)	0.64 (0.52–0.81), < 0.001	0.81 (0.62–1.05), 0.115
**Gravida**			
1	2.59 (2.37–2.84)	1.00 (referent)	1.00 (referent)
2	1.93 (1.71–2.17)	0.74 (0.63–0.86), < 0.001	0.78 (0.65–0.93), 0.006
3	2.11 (1.72–2.59)	0.81 (0.65–1.02), 0.070	0.81 (0.62–1.06), 0.129
≥ 4	3.57 (2.71–4.69)	1.39 (1.03–1.88), 0.031	1.31 (0.93–1.86), 0.125
**Pregnancy registered with**			
Primary Healthcare Centre	2.41 (2.24–2.59)	1.00 (referent)	1.00 (referent)
Other establishment	1.96 (1.67–2.31)	0.81 (0.68–0.97), 0.025	0.88 (0.73–1.06), 0.193
**Blood pressure was checked**			
Yes	2.32 (2.18–2.48)	1.00 (referent)	1.00 (referent)
No	2.62 (1.25–5.40)	1.13 (0.53–2.40), 0.748	0.84 (0.31–2.33), 0.741
**Weight was checked**			
Yes	2.32 (2.18–2.48)	1.00 (referent)	1.00 (referent)
No	2.66 (1.27–5.48)	1.15 (0.54–2.44), 0.717	0.87 (0.32–2.38), 0.787
**Blood group was checked**			
Yes	2.04 (1.87–2.23)	1.00 (referent)	1.00 (referent)
No	2.79 (2.53–3.07)	1.37 (1.20–1.57), < 0.001	1.21 (1.04–1.40), 0.012
**Haemoglobin level was checked**			
Yes	2.29 (2.15–2.45)	1.00 (referent)	1.00 (referent)
No	3.32 (2.43–4.54)	1.46 (1.05–2.04), 0.024	1.28 (0.88–1.87), 0.193
**Urine sugar was checked**			
Yes	2.32 (2.16–2.49)	1.00 (referent)	1.00 (referent)
No	2.40 (2.00–2.86)	1.04 (0.85–1.26), 0.729	1.26 (0.76–2.07), 0.371
**Urine protein was checked**			
Yes	2.33 (2.17–2.51)	1.00 (referent)	1.00 (referent)
No	2.28 (1.92–2.71)	0.98 (0.81–1.18), 0.815	0.71 (0.44–1.15), 0.165
**Number of antenatal care visit**			
< 4	2.50 (2.31–2.70)	1.00 (referent)	1.00 (referent)
≥ 4	1.99 (1.76–2.25)	0.79 (0.68–0.92), 0.002	0.90 (0.77–1.05), 0.182
**Place of delivery**			
Healthcare facility	2.16 (2.00–2.32)	1.00 (referent)	1.00 (referent)
Non-healthcare facility	3.55 (3.05–4.13)	1.67 (1.40–1.99), < 0.001	1.63 (1.31–2.02), < 0.001
**Type of delivery**			
Normal	2.45 (2.28–2.64)	1.00 (referent)	1.00 (referent)
Caesarean/forceps	1.81 (1.53–2.14)	0.73 (0.61–0.88), 0.001	0.96 (0.79–1.16), 0.663
**Delivery conducted by**			
Trained personnel	2.27 (2.12–2.42)	1.00 (referent)	1.00 (referent)
Untrained personnel	4.08 (3.12–5.33)	1.84 (1.38–2.45), < 0.001	1.24 (0.88–1.74), 0.225
**Number of iron-and-folic acid consumption**			
< 100	2.78 (2.58–3.00)	1.00 (referent)	1.00 (referent)
≥ 100	1.57 (1.38–1.79)	0.56 (0.48–0.65), < 0.001	0.70 (0.59–0.82), < 0.001
**Sex of the child**			
Male	2.63 (2.41–2.86)	1.00 (referent)	1.00 (referent)
Female	2.00 (1.81–2.22)	0.76 (0.66–0.87), < 0.001	0.71 (0.62–0.82), < 0.001
**Birthweight status**			
Low birthweight	5.63 (5.12–6.18)	1.00 (referent)	1.00 (referent)
Normal	1.53 (1.39–1.67)	0.26 (0.23–0.30), < 0.001	0.26 (0.23–0.30), < 0.001
**Overall**	2.33 (2.18–2.48)		

*CI* confidence interval, *OR* odds ratio, *p* level of significance.

## Discussion

Using a ten-years prospective cohort data from the BIRPOP-HDSS located in the state of West Bengal, India, this study examined the association of use of ANC and delivery care services with maternal and infant mortality. The findings revealed that ≥ 100 iron-and-folic acid (IFA) tablet/equivalent syrup consumption during pregnancy had a protective association with both maternal and infant death. No check-up of maternal blood-group during the pregnancy was associated with increased odds of the death of infants. Caesarean/forceps delivery and delivery conducted by untrained personnel were also found associated with increased odds of maternal mortality.

Findings revealed that ≥ 100 IFA consumption has a protective association with maternal and infant mortality. Consumption of IFA tablet/equivalent syrup during pregnancy is considered as an effective intervention to mitigate maternal and child mortality^40^. The National Iron Plus Initiative (NIPI) of India recommends that every pregnant woman should consume 100 mg of elemental iron and 500 mcg of folic acid daily for 100 days during pregnancy. Also, the current Anaemia*-Mukt Bharat* (Anaemia-Free India), a strategy launched in 2018, aims to achieve the targets of POSHAN *Abhiyaan* (POSHAN: Prime Minister’s Overarching Scheme for Holistic Nutrition Mission), has taken every possible initiative to attain reduction of prevalence of maternal and child anaemia by three percentage points per year^41^. Therefore, finding of this study is consistent with the recommendation of IFA consumption to increase maternal and infant survival. Earlier studies conducted in India^42–46^ have discussed the coverage of IFA and possible pathways to improve maternal and child survival in India. A recent study^37^ in India analysed nationally representative data and showed that consumption of IFA by pregnant mothers could prevent neonatal mortality.

The results suggest that unchecked blood-group during pregnancy was associated with increased odds of infant mortality. During pregnancy, women could expect a range of physiological changes in their haematological parameter which requires constant monitoring for a better maternal and child health outcome ^47^. Although detailed information on blood group is not available in this study, studies^48^ indicate that failure to do a routine blood check-up during pregnancy could be fatal for both prospective mothers and their babies. Checking of blood group is essential during pregnancy as a blood transfusion might be required (due to loss of blood, either in a surgery, delivery, or accident) to save the mother and the baby. Also, depending on mother’s blood type, babies may develop some life threatening complications^49^.

Findings from multivariable analysis further suggested that maternal mortality was higher among women who underwent caesarean section delivery. Earlier studies^50,51^ have discussed the pervasive and increased burden of caesarean section delivery in India and this trend could be detrimental for maternal health. Delivery through caesarean sections could lead to significant and sometimes permanent complications, disability or death, particularly in settings that lack the facilities and/or capacity to properly conduct safe surgery and treat the surgical complications arising from it. Caesarean section should ideally be undertaken only when medically necessary^52^. A recent study ^53^ empirically demonstrated that elective caesarean section delivery could lead to excess weight and possibly reduced linear growth at one year of age in children in India. Generally, women or doctors in rural India opt for caesarean/forceps delivery if there are some pregnancy or birth complications and any pregnancy or birth complication can also be a reason for maternal death. Since the information on experience of pregnancy/birth complications is not available, this study calls for further research of this relationship.

In addition, multivariable regression results indicate that delivery conducted by untrained personnel could lead to maternal death. This finding is consistent with previous studies^54,55^, which document that delivery assisted by health professionals are safe and all efforts should be made to deliver the baby at the health facility only, and if it is not possible to conduct a delivery at the health facility, the delivery should be conducted by a trained health professional. Evidence from India^56^ suggests that well trained and dedicated personnel in home-based delivery care could also be effective in improving maternal and child health outcome.

Findings of this study should be interpreted considering its possible limitations. First, not all potential predictors of maternal and infant mortality were available to control in the multivariable logistic regression analysis. For example, further information on social indicators (for example: religion, caste, wealth, parental education, and parental occupation); information on role of the *Anganwadi* (synonymous to courtyard shelter), type of health facility (public/private), distance to the nearest health facility, and transport facility used for reaching the hospital at the time of delivery, breastfeeding status of children; detailed information on measurements taken during ANC; and detailed information on post-natal care provided to mother and children could have been helpful in ushering further details on importance of ANC and delivery care in mitigating maternal and infant mortality. Second, although all pregnant mothers were paid a visit at least once a month by the surveyor to gather necessary information on ANC and delivery care services accessed by them, there is always a chance of recall errors and/or social desirability bias in reporting. Third, this study does not analyse a causal relationship between ANC and delivery care and maternal and infant mortality, thus caution in interpretation of results is urged. Fourth and final, findings of this study are applicable to the BIRPOP-HDSS population only, thus generalization of the study findings is not encouraged. Despite these limitations, this study is first of its kind that uses 10 years’ data on pregnancies and ANC and delivery care services and investigates how they are linked with improving maternal and infant mortality.

Findings from this study emphasizes on increasing coverage and consumption of IFA tablets/equivalent syrup. With improved ANC and delivery services, increased uptake of all types of ANC and delivery care services are equally important for improvement in maternal and child survival in rural India. However, two urgent measures should be prioritized to reduce maternal and infant mortality. First, although India is celebrating the increased uptake of ≥ 100 IFA (from 30.3% during 2015–16 to 44.1% during 2019–21)^18^, it still needs an appropriate method of anaemia measurement. More research is needed on required dose of iron and long-term effect of iron overdose among pregnant mothers^46^. Second, the Government of India should more stringently monitor and reprehend unnecessary caesarean section delivery, as its high and growing prevalence is concerning^57^.

## Data Availability

The datasets generated and/or analysed for the current study are available from the corresponding author upon reasonable request.

## References

[1] GBD 2015 Maternal Mortality Collaborators. Global, regional, and national levels of maternal mortality, 1990–2015: A systematic analysis for the Global Burden of Disease Study 2015. *Lancet* **388**, 1775–1812 (2016). 10.1016/S0140-6736(16)31470-2 PMC5224694 27733286

[2] GBD 2019 Under-5 Mortality Collaborators. Global, regional, and national progress towards Sustainable Development Goal 3.2 for neonatal and child health: all-cause and cause-specific mortality findings from the Global Burden of Disease Study 2019. *Lancet* **398**, 870–905 (2021). 10.1016/S0140-6736(21)01207-1 PMC8429803 34416195

[3] Office of the Registrar General. *Special Bulletin on Maternal Mortality in India 2016–18*. (Sample Registration System, Government of India, 2020).

[4] Ministry of Health and Family Welfare. Status of IMR and MMR in India. *Press Information Bureau*. https://pib.gov.in/PressReleaseIframePage.aspx?PRID=1796436 (2022).

[5] Singh L (2022). Coverage of quality maternal and newborn healthcare services in India: Examining dropouts, disparity and determinants. Ann. Glob. Health.

[6] Leventhal DG (2021). Delivery channels and socioeconomic inequalities in coverage of reproductive, maternal, newborn, and child health interventions: Analysis of 36 cross-sectional surveys in low-income and middle-income countries. Lancet Glob. Health.

[7] Black, R. E., Walker, N., Laxminarayan, R. & Temmerman, M. Reproductive, maternal, newborn, and child health: Key messages of this volume. in *Reproductive, Maternal, Newborn, and Child Health: Disease Control Priorities *(Black, R.E. et al. eds.). 3rd edn. Vol. 2. (The International Bank for Reconstruction and Development/The World Bank, 2016). 27227235

[8] World Health Organization. *WHO Recommendations on Antenatal Care for a Positive Pregnancy Experience*. (World Health Organization, 2016). 28079998

[9] Willcox ML (2020). Death audits and reviews for reducing maternal, perinatal and child mortality. Cochrane Database Syst. Rev..

[10] Lassi ZS, Bhutta ZA (2015). Community-based intervention packages for reducing maternal and neonatal morbidity and mortality and improving neonatal outcomes. Cochrane Database Syst. Rev..

[11] Berhan Y, Berhan A (2014). Antenatal care as a means of increasing birth in the health facility and reducing maternal mortality: A systematic review. Ethiop. J. Health Sci..

[12] Neupane S, Doku DT (2019). Association of the quality of antenatal care with neonatal mortality: Meta-analysis of individual participant data from 60 low- and middle-income countries. Int. Health.

[13] Doku DT, Neupane S (2017). Survival analysis of the association between antenatal care attendance and neonatal mortality in 57 low- and middle-income countries. Int. J. Epidemiol..

[14] Islam MA, Tabassum T (2021). Does antenatal and post-natal program reduce infant mortality? A meta-analytical review on 24 developing countries based on Demographic and Health Survey data. Sex. Reprod. Healthc..

[15] Wondemagegn AT, Alebel A, Tesema C, Abie W (2018). The effect of antenatal care follow-up on neonatal health outcomes: A systematic review and meta-analysis. Public Health Rev..

[16] Tekelab T, Chojenta C, Smith R, Loxton D (2019). The impact of antenatal care on neonatal mortality in sub-Saharan Africa: A systematic review and meta-analysis. PLoS ONE.

[17] World Health Organization. *WHO Safe Childbirth Checklist Implementation Guide: Improving the Quality of Facility-Based Delivery for Mothers and Newborns*. (WHO, 2015).

[18] International Institute for Population Sciences (IIPS) and ICF. *National Family Health Survey (NFHS-5), 2019–21: India*. (IIPS, 2021).

[19] Kumar G (2019). Utilisation, equity and determinants of full antenatal care in India: Analysis from the National Family Health Survey 4. BMC Pregnancy Childb..

[20] Singh L (2019). Measuring quality of antenatal care: A secondary analysis of national survey data from India. BJOG.

[21] Singh P, Singh KK, Singh P (2021). Maternal health care service utilization among young married women in India, 1992–2016: Trends and determinants. BMC Pregnancy Childb..

[22] Goli S, Puri P, Salve PS, Pallikadavath S, James KS (2022). Estimates and correlates of district-level maternal mortality ratio in India. PLOS Glob. Public Health.

[23] Sanneving L, Trygg N, Saxena D, Mavalankar D, Thomsen S (2013). Inequity in India: The case of maternal and reproductive health. Glob. Health Action.

[24] Bhatia M (2021). Pro-poor policies and improvements in maternal health outcomes in India. BMC Pregnancy Childb..

[25] Horwood G, Opondo C, Choudhury SS, Rani A, Nair M (2020). Risk factors for maternal mortality among 1.9 million women in nine empowered action group states in India: Secondary analysis of Annual Health Survey data. BMJ Open.

[26] Singh A, Pallikadavath S, Ram F, Alagarajan M (2014). Do antenatal care interventions improve neonatal survival in India?. Health Policy Plan..

[27] Kuhnt J, Vollmer S (2017). Antenatal care services and its implications for vital and health outcomes of children: Evidence from 193 surveys in 69 low-income and middle-income countries. BMJ Open.

[28] Montgomery AL, Ram U, Kumar R, Jha P (2014). Million Death Study Collaborators. Maternal mortality in India: Causes and healthcare service use based on a nationally representative survey. PLoS ONE.

[29] Horwood G, Opondo C, Choudhury SS, Rani A, Nair M (2020). Risk factors for maternal mortality among 1.9 million women in nine empowered action group states in India: Secondary analysis of Annual Health Survey data. BMJ Open.

[30] Lee HY (2022). The association between institutional delivery and neonatal mortality based on the quality of maternal and newborn health system in India. Sci. Rep..

[31] Das U, Chaplot B, Azamathulla HM (2021). The role of place of delivery in preventing neonatal and infant mortality rate in India. Geographies.

[32] Altman R, Sidney K, De Costa A, Vora K, Salazar M (2017). Is institutional delivery protective against neonatal mortality among poor or tribal women? a cohort study from Gujarat, India. Matern. Child Health J..

[33] Ghosh S (2015). Health & demographic surveillance system profile: The Birbhum population project (Birbhum HDSS). Int. J. Epidemiol..

[34] Rai RK (2020). Non-communicable diseases are the leading cause of mortality in rural Birbhum, West Bengal, India: A sex-stratified analysis of verbal autopsies from a prospective cohort, 2012–2017. BMJ Open.

[35] Ministry of Health and Family Welfare. *A Strategic Approach to Reproductive, Maternal, Newborn**, **Child and Adolescent Health (RMNCH+A) in India*. (Government of India, 2013).

[36] Rai, R. K. Age of marriage and nutritional status among women aged 15–24 years: A nationally representative quasi-experimental study in India. in *Four Essays on Health and Nutrition of Indian Women and Children (Unpublished PhD Thesis) *(Rai, R. K. eds.). (Faculty of Economic Sciences, Georg-August-Universität Göttingen, 2022).

[37] Rai RK, De Neve JW, Geldsetzer P, Vollmer S (2022). Maternal iron-and-folic-acid supplementation and its association with low-birth weight and neonatal mortality in India. Public Health Nutr..

[38] Chatterjee S, Hadi AS (2006). Regression Analysis by Example.

[39] StataCorp. *Stata Statistical Software: Release 14*. (StataCorp LP, 2015).

[40] Ministry of Health and Family Welfare. *Guideline for Control of Iron Deficiency Anaemia.* (Adolescent Division, MoHFW, Government of India, 2013).

[41] Joe W (2022). Coverage of iron and folic acid supplementation in India: Progress under the *Anemia*
*Mukt* Bharat strategy 2017–20. Health Policy Plan..

[42] Rai RK (2022). Iron-and-folic-acid supplementation among adolescents (aged 10–19 years) in two North Indian States, 2015–2016: A sex-stratified analysis. Public Health Nutr..

[43] Singh PK (2020). Public health interventions to improve maternal nutrition during pregnancy: A nationally representative study of iron and folic acid consumption and food supplements in India. Public Health Nutr..

[44] Sudfeld CR, Rai RK, Barik A, Valadez JJ, Fawzi WW (2020). Population-level effective coverage of adolescent weekly iron and folic acid supplementation is low in rural West Bengal, India. Public Health Nutr..

[45] Rai RK, Fawzi WW, Barik A, Chowdhury A (2018). The burden of iron-deficiency anaemia among women in India: how have iron and folic acid interventions fared? WHO South East Asia. J. Public Health.

[46] Rai RK (2022). Shooting shadows: India's struggle to reduce the burden of anaemia. Br. J. Nutr..

[47] Chandra S, Tripathi AK, Mishra S, Amzarul M, Vaish AK (2012). Physiological changes in hematological parameters during pregnancy. Indian J. Hematol. Blood Transfus..

[48] Mavalankar DV (2009). Maternal health in Gujarat, India: A case study. J. Health Popul. Nutr..

[49] Pegoraro V (2020). Hemolytic disease of the fetus and newborn due to Rh(D) incompatibility: A preventable disease that still produces significant morbidity and mortality in children. PLoS ONE.

[50] Bhatia M, Banerjee K, Dixit P, Dwivedi LK (2020). Assessment of variation in cesarean delivery rates between public and private health facilities in India from 2005 to 2016. JAMA Netw. Open.

[51] Lee HY, Kim R, Oh J, Subramanian SV (2021). Association between the type of provider and Cesarean section delivery in India: A socioeconomic analysis of the National Family Health Surveys 1999, 2006, 2016. PLoS ONE.

[52] World Health Organization. *WHO Statement on Caesarean Section Rates*. (WHO, 2015).

[53] Babu GR (2021). Delivery mode and altered infant growth at 1 year of life in India. Pediatr. Res..

[54] Meh C (2022). Trends in maternal mortality in India over two decades in nationally representative surveys. BJOG.

[55] Rai RK, Tulchinsky TH (2015). Addressing the sluggish progress in reducing maternal mortality in India. Asia Pac. J. Public Health.

[56] Rasaily R (2020). Effect of home-based newborn care on neonatal and infant mortality: A cluster randomised trial in India. BMJ Glob. Health.

[57] Singh PK, Rai RK, Singh S, Singh L (2018). Rising caesarean births - A growing concern. Econ. Polit. Wkly..

